# Spilled but Not Forgotten: A Retained Gallstone Leading to Colonic Fistula Formation 

**DOI:** 10.7759/cureus.80556

**Published:** 2025-03-14

**Authors:** Abeer Alzuabi, Warda Anam, Mohammed Alblooshi, Shadi Al-Bahri, Guido H Mannaerts

**Affiliations:** 1 Anesthesia and Intensive Care, Al Qassimi Hospital, Sharjah, ARE; 2 General Surgery, Sheikh Shakhbout Medical City (SSMC), Abu Dhabi, ARE; 3 General Surgery, Tawam Hospital, Al-Ain, ARE; 4 General Surgery, Sheikh Tahnoon Medical City, Al-Ain, ARE; 5 Surgery, United Arab Emirates University (UAEU) College of Medicine and Health Sciences, Al-Ain, ARE; 6 General Surgery, Tawam Hospital, Al-Ain, USA

**Keywords:** colonic fistula, gallbladder perforation, intra-abdominal abscess, laparoscopic cholecystectomy complications, spilled gallstones

## Abstract

Laparoscopic cholecystectomy (LC) is a routinely performed procedure for symptomatic cholelithiasis, known for its minimal invasiveness and favorable outcomes; however, intraoperative gallbladder perforation and subsequent stone spillage can lead to rare but serious complications such as abscess formation and fistulization. A 38-year-old male patient with a history of sickle cell disease and prior LC presented multiple times with recurrent right upper quadrant (RUQ) pain and sepsis, and over the course of two years, imaging and endoscopic evaluations revealed persistent subhepatic abscesses with a fistulous tract involving the ascending colon. Intraoperative exploration ultimately uncovered a retained gallstone serving as the nidus of this chronic inflammatory process, and a laparoscopic right hemicolectomy with the removal of the stone was performed, leading to the resolution of the patient’s symptoms. Although many retained gallstones remain clinically silent, a fraction present late with abscesses or fistulas, complicating diagnosis and increasing morbidity; therefore, comprehensive imaging studies, including CT scans and colonoscopy, are essential for identifying potential complications, and definitive surgical intervention is often required to remove the affected bowel segment and the offending stone. This case underscores the importance of meticulous intraoperative technique and vigilance in patients who develop unexplained or recurrent abdominal sepsis after LC, highlighting that early recognition of this rare complication can guide appropriate management and ultimately reduce the risk of severe morbidity.

## Introduction

Laparoscopic cholecystectomy (LC) is widely recognized as the gold-standard procedure for the treatment of cholelithiasis, having largely supplanted open cholecystectomy due to its well-established benefits of reduced postoperative pain, shorter hospital stays, and faster return to normal activities [1]. Despite its favorable safety profile, LC is not without complications. Gallbladder perforation during LC has been reported to occur in 36.1% of patients, often leading to the spillage of stones into the abdominal cavity [2].

Although many of these lost stones remain clinically silent, a small subset can precipitate serious sequelae such as abscess formation, adhesions, and, in rare cases, fistulization to adjacent structures [3]. The risk is heightened when multiple gallstones, pigmented stones, or evidence of acute cholecystitis is present, as these factors may predispose patients to more severe inflammatory responses [4]. The latency period for clinical manifestations can extend from months to years postoperatively, and early detection of retained stones can be challenging [1].

This case report describes a rare but significant complication wherein a single spilled stone from a perforated gallbladder led to recurrent subhepatic abscesses and the eventual formation of a colonic fistula. Given the potential for diagnostic delay and recurrent morbidity, it is crucial that surgeons maintain a high index of suspicion for this complication when patients present with persistent or unexplained abdominal symptoms following LC. By highlighting this scenario, we hope to underscore the importance of meticulous surgical technique, vigilant postoperative follow-up, and early use of advanced imaging when indicated. Furthermore, in accordance with the Surgical Case Report (SCARE) guidelines, we provide a detailed account of this case to facilitate structured and transparent reporting of such a rare surgical complication [5].

## Case presentation

A 38-year-old male patient with sickle cell disease and a history of splenectomy at the age of eight presented in May 2017 with right upper quadrant (RUQ) pain, jaundice, and deranged liver function tests (LFTs). Diagnostic imaging and laboratory workup were consistent with choledocholithiasis. An endoscopic retrograde cholangiopancreatography (ERCP) revealed an impacted stone at the ampulla, and sphincterotomy with stone extraction was performed.

Initial presentation and surgical intervention

In December 2018, the patient returned with acute RUQ pain suggestive of acute cholecystitis. Despite being offered admission, he initially signed out against medical advice to attempt outpatient antibiotic therapy. One month later, he was readmitted with persistent RUQ pain and acute kidney injury. Laboratory findings again showed elevated inflammatory markers and LFT abnormalities. An urgent magnetic resonance cholangiopancreatography (MRCP) and repeat ERCP excluded choledocholithiasis or intrahepatic ductal dilation.

On January 3, 2019, the patient underwent LC. After identifying the critical view of safety, the cystic duct was clearly visualized and doubly clipped before transection. However, the gallbladder was markedly distended, edematous, and gangrenous, leading to a perforation of the gallbladder wall and the spillage of purulent fluid and gallstones into the peritoneal cavity. No additional suture was deemed necessary for the cystic duct stump, but extensive irrigation with normal saline was performed to remove any contaminated fluid or residual stones, and a drain was placed near the gallbladder fossa; it remained in situ for five days and was removed on postoperative day six. Postoperatively, the patient’s inflammatory markers trended downward, and he was discharged on postoperative day six (Table 1).

**Table 1:** Laboratory results of the patient around the time of laparoscopic cholecystectomy Reference ranges: total bilirubin: 5.0–21.0 µmol/L; direct bilirubin: 0–7.0 µmol/L; white blood cell (WBC): 4.0–11.0 ×10^9/L; C-reactive protein (CRP): <5 mg/L

Date	Total bilirubin (µmol/L)	Direct bilirubin (µmol/L)	WBC (×10^9/L)	CRP (mg/L)
30/01/2019	32.9	16.0	11.6	–
31/01/2019	64.4	32.9	26.9	100.2
02/02/2019	51.1	35.7	19.1	201.6
03/02/2019	42.4	33.2	11.8	136.8

Laboratory profiles around surgery

Recurrent Abscesses and Workup

Over the ensuing year, the patient developed multiple episodes of RUQ pain and fever, necessitating repeated emergency department visits. In August 2019, an ultrasound of the abdomen demonstrated a 4.4 × 3.7 cm abscess in segment VI of the liver. Image-guided drainage yielded purulent fluid, which was sent for microbiological culture, and intravenous antibiotics were administered based on the culture sensitivities once results became available. Despite initial clinical improvement, the patient’s symptoms recurred in January 2020. Repeat labs showed ongoing elevations in bilirubin and inflammatory markers (Table 2).

**Table 2:** Laboratory results during readmission with recurrent right upper quadrant (RUQ) pain and fever Reference ranges: total bilirubin: 5.0–21.0 µmol/L; direct bilirubin: 0–7.0 µmol/L; gamma-glutamyl transferase (GGT): 9–48 U/L; white blood cell (WBC): 4.0–11.0 ×10^9/L; C-reactive protein (CRP): <5 mg/L

Date	Total bilirubin (µmol/L)	Direct bilirubin (µmol/L)	GGT (U/L)	WBC (×10^9/L)	CRP (mg/L)
12/01/2020	39.8	21.1	–	10.5	–
13/01/2020	32.6	20.8	110	10.2	106.5

**Figure 1:**
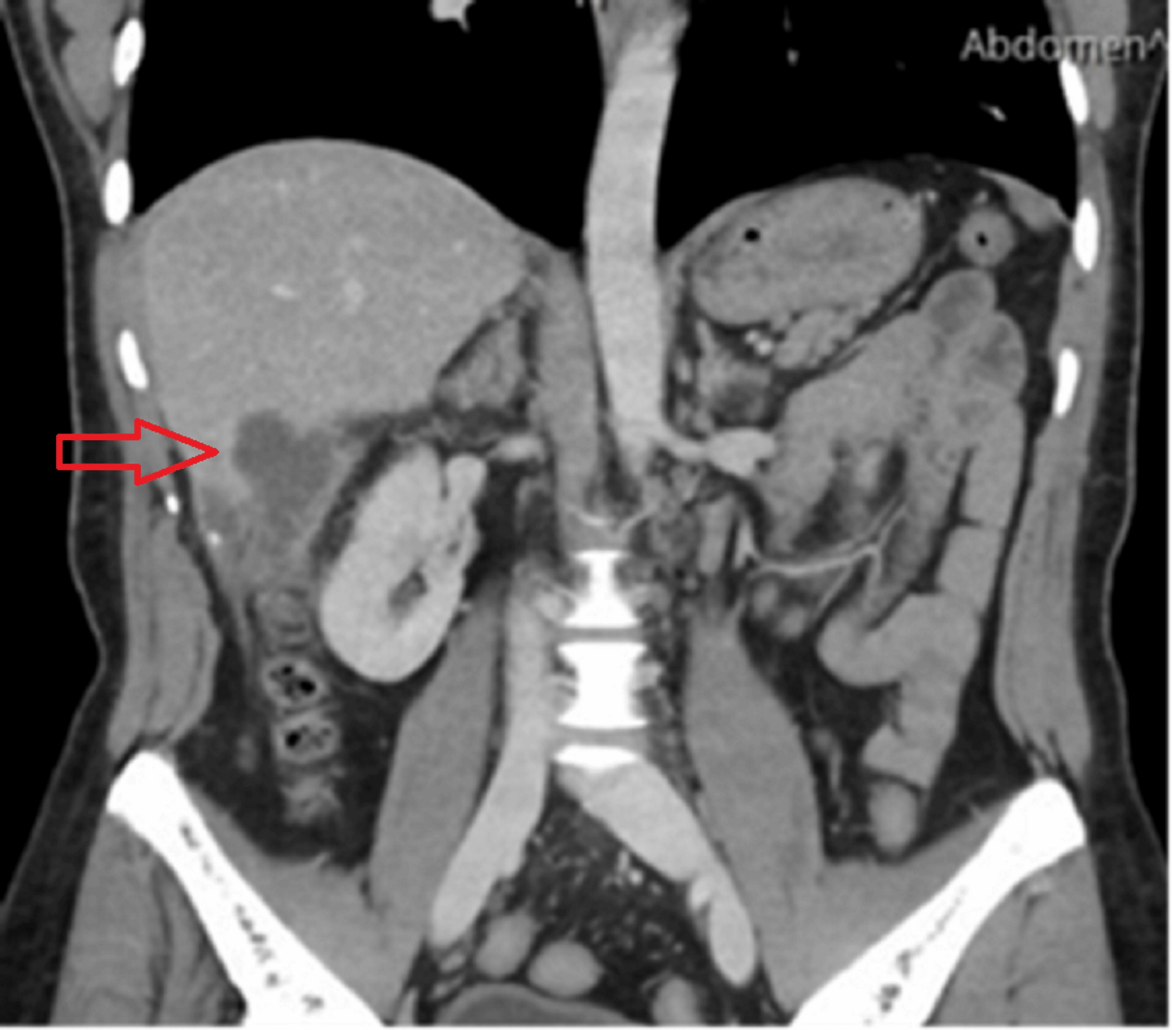
A CT scan of the abdomen demonstrating a subhepatic abscess measuring 2.5 × 4.0 cm with extension to the ascending colon The lower density area, as indicated by the arrow, corresponds to a fluid collection consistent with abscess formation.

A contrast-enhanced computed tomography (CT) scan of the abdomen revealed a 2.5 × 4.0 cm fluid collection in the subhepatic region, extending into the ascending colon with significant fat stranding (Figure 1). Conservative management with antibiotics was initially pursued.

Colonoscopic Findings

**Figure 2:**
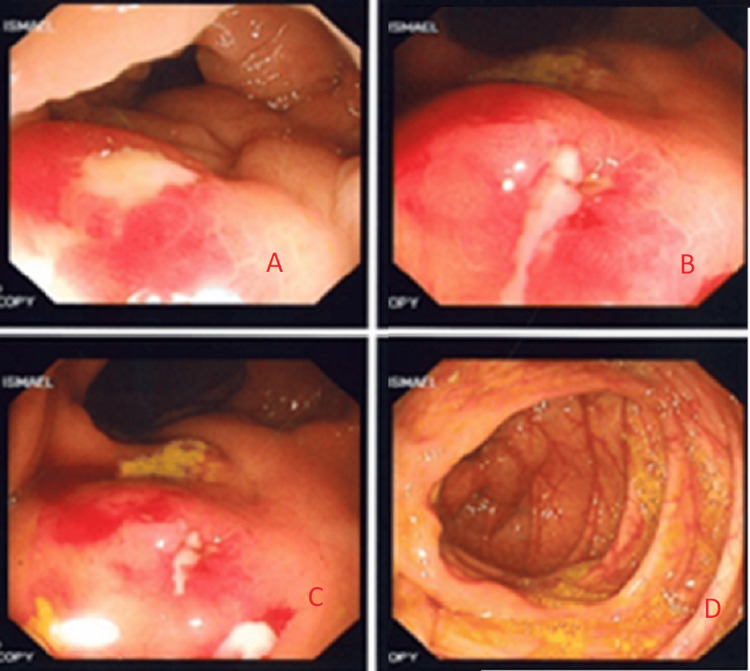
Colonoscopy images showing the fistulous tract near the hepatic flexure (wider view) Colonoscopic views from the transverse colon near the hepatic flexure; (A–C) demonstrate erythematous, inflamed mucosa (red areas) corresponding to the suspected fistulous regions at the hepatic flexure, indicating pronounced inflammatory changes. (D) shows normal-appearing colonic mucosa in the transverse colon for comparison, with no evidence of inflammation or fistulization.

On January 15, 2020, a colonoscopy identified a fistulous opening near the hepatic flexure (Figure 2). This correlated with the CT findings suggesting an ongoing connection between the hepatic flexure and the abscess cavity. A repeat MRCP confirmed the persistent abscess (now measuring approximately 2.38 × 2.08 cm) and a tract communicating directly with the ascending colon.

Surgical Exploration

**Figure 3:**
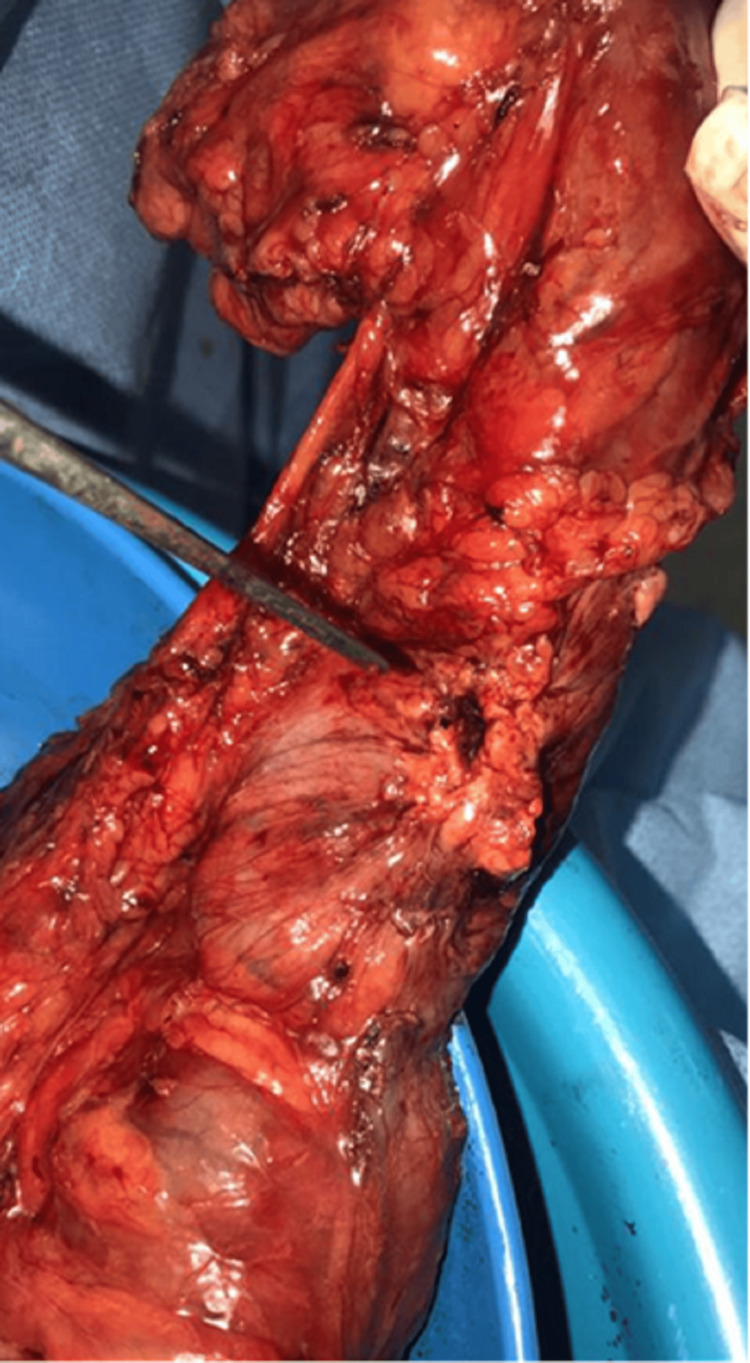
Intraoperative view of dense adhesions at the hepatic flexure

**Figure 4:**
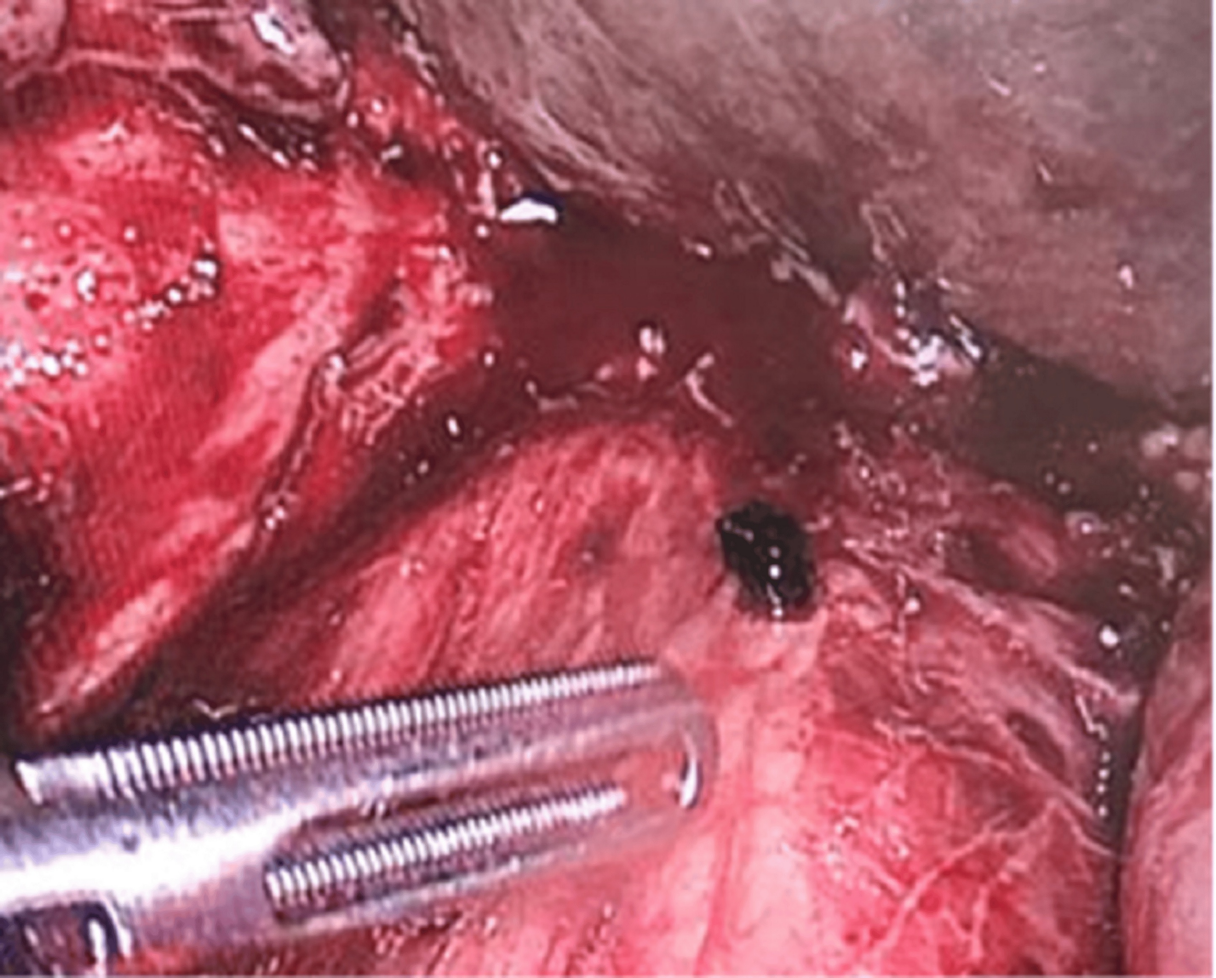
Intraoperative finding of the retained stone within inflammatory tissue

**Figure 5:**
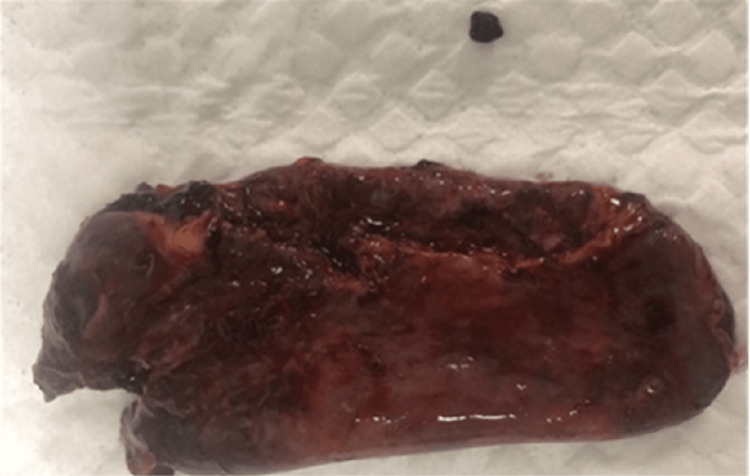
A closer view of the retained stone in relation to the colon

Given the recurrent infections and established colonic fistula, the patient was scheduled for a laparoscopic right hemicolectomy on January 22, 2020. Intraoperative exploration disclosed dense adhesions at the hepatic flexure. Upon mobilizing the colon, frank purulent material and fibrinous exudates were observed around the fistulous tract (Figure 3). Significantly, a retained gallstone was discovered embedded in the inflammation, confirming it as the nidus for chronic sepsis and fistula formation (Figures 4, 5). 

A laparoscopic right hemicolectomy with primary ileocolic anastomosis was performed (Appendix A). This location aligns closely with the content of the video, ensuring that readers can directly relate the text to the surgical footage.

**Figure 6:**
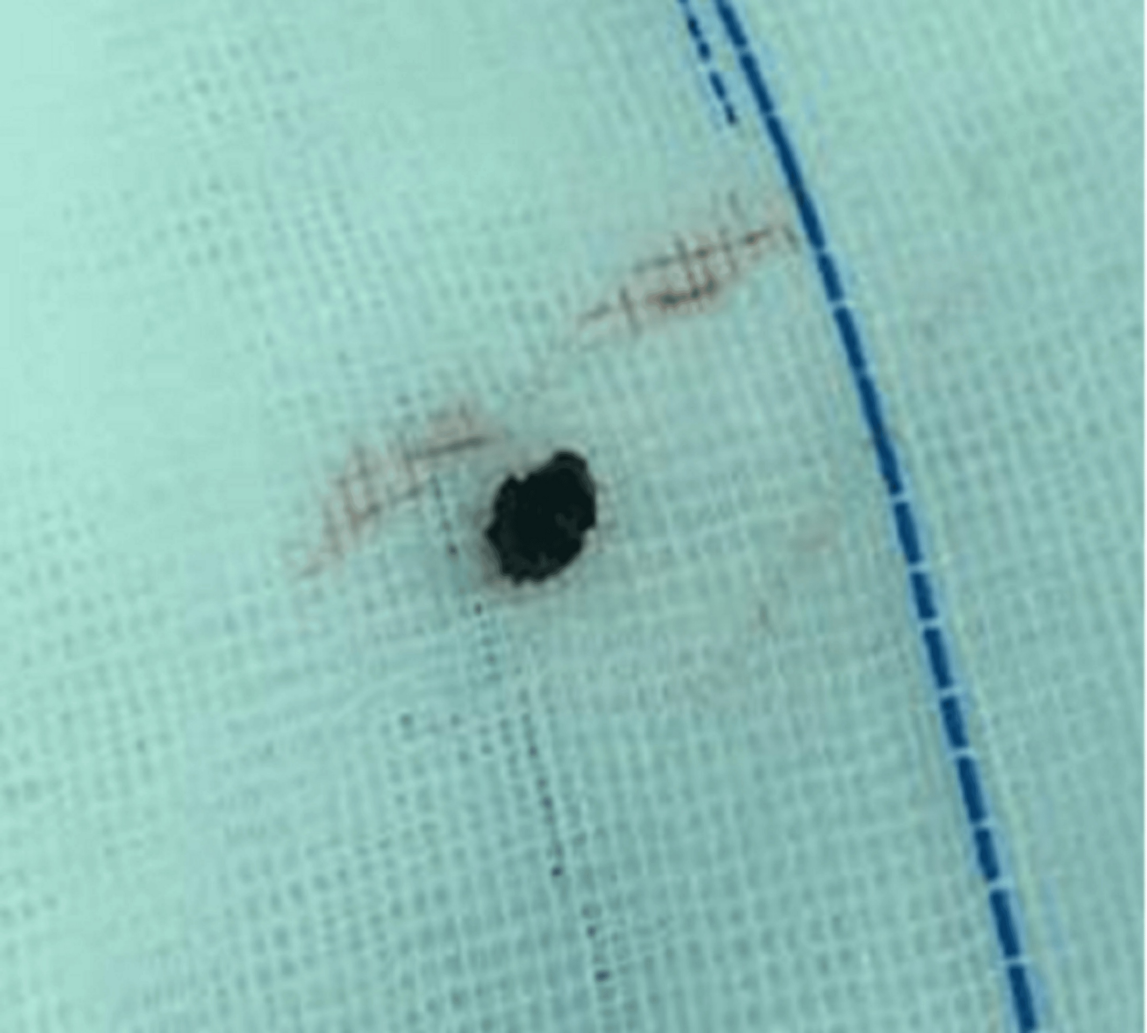
Gallstone retrieved from the fistulous tract

A right hemicolectomy with primary ileocolic anastomosis was performed. The resected specimen revealed an external fistulous opening, and a gallstone was identified within the tract serving as the nidus of chronic infection (Figure 6). The stone was removed intact. Postoperatively, the patient convalesced uneventfully, with no further sepsis or abscess formation at follow-up.

Overview of Related Complications

Retained or spilled gallstones are among the rare complications of laparoscopic cholecystectomy and may present with varied and delayed manifestations. A large retrospective analysis of 9,542 laparoscopic cholecystectomies by Duca et al. provides detailed insights into early complications and how they were managed [6]. This overview highlights various treatment modalities, ranging from conservative to minimally invasive to open surgery depending on the specific complication encountered.

Out of the 9,542 laparoscopic cholecystectomies reviewed, a total of 90 major complications were identified. These consisted of 34 bile leaks (19 managed conservatively, 11 with endoscopic sphincterotomy, and four requiring open surgery), 20 choleperitoneum events (five resolved laparoscopically and 15 with an open procedure), 15 instances of postoperative hemorrhage (seven handled conservatively, four laparoscopically, and four with an open approach), 10 subhepatic abscesses (seven treated laparoscopically and three through open intervention), and 11 retained bile duct stones (all managed by endoscopic sphincterotomy). Overall, 26 (28.9%) of the 90 complications were resolved by conservative measures, 38 (42.2%) by minimally invasive techniques, and 26 (28.8%) required open surgery [6]. These findings underscore that while a significant proportion of complications can be resolved non-operatively or through less invasive strategies, open surgical intervention remains essential in certain complex situations.

Taken together, this patient’s clinical course highlights the potential for spilled gallstones to generate chronic inflammatory processes and fistulization. Thorough irrigation, surgical vigilance during gallbladder extraction, and heightened clinical suspicion in postoperative patients with unexplained abscesses are crucial for reducing the likelihood of such rare but significant complications.

## Discussion

Laparoscopic cholecystectomy is undeniably the gold-standard treatment for cholelithiasis, yet inadvertent gallbladder perforation and stone spillage occur in approximately 0.2% to 2.3% of all procedures [7]. While many of these stones remain clinically quiescent, a notable proportion can lead to significant complications such as adhesions, sinus tract formation, abscesses, or, more rarely, fistulization into adjacent structures [8]. The clinical diagnosis of these rare but serious sequelae can be delayed by months or even years owing to an often indolent inflammatory process.

Pathophysiology of Spilled Stones

In the setting of acute or gangrenous cholecystitis, as in this case, the friable gallbladder wall is more prone to perforation. When stones are dropped into the peritoneal cavity, they can become embedded in the omentum or adhere to inflamed tissue. Over time, these retained gallstones may serve as a nidus for persistent infection or chronic inflammatory changes, culminating in abscess formation. If the inflammatory process erodes into adjacent organs, a fistulous tract may result [9]. Although entero-biliary fistulas (particularly cholecystocolonic fistulas) have been well-documented in the setting of gallstone disease, a fistula caused by a single retained gallstone post-laparoscopic surgery is far more unusual [10].

Diagnostic Challenges

The latency period between the initial surgery and the manifestation of symptoms can vary significantly. Patients may present with vague, nonspecific symptoms such as intermittent abdominal pain, low-grade fever, or malaise [7]. This delayed, insidious course often leads clinicians to investigate other, more common etiologies of recurrent abscesses. Cross-sectional imaging, particularly contrast-enhanced CT, remains pivotal for detecting intra-abdominal fluid collections and potentially identifying retained gallstones. However, stones can sometimes be missed if they are small or if their density is similar to surrounding tissues [11]. Complementary modalities, including MRCP and colonoscopy, are especially useful when clinical suspicion for a fistula or ductal abnormality is high.

In this case, a fistulous connection to the ascending colon was eventually visualized during colonoscopy. Such a finding underscores the importance of comprehensive endoscopic evaluations when imaging suggests loculated collections abutting hollow viscera. As reported by the Southern Surgeons Club (2008), a meticulous perioperative technique comprising careful dissection, the use of retrieval bags, and thorough irrigation can substantially mitigate the risk of missed stones or septic complications [12].

Risk Factors and Prevention Strategies

Multiple literature reviews suggest that acute inflammation, large gallstones, pigmented stones, and obesity are contributory risk factors for spillage leading to complications [7,9]. Prolonged operative times, distorted anatomy, and extensive adhesions further increase the likelihood of intraoperative perforation. Preventive strategies include the routine use of retrieval pouches placing the gallbladder in a specimen bag prior to removal to minimize the risk of stone loss avoidance of excessive traction since overly aggressive traction on the gallbladder can lead to perforation and stone extrusion, copious irrigation after any suspicion of perforation, as thorough irrigation with normal saline reduces the bacterial load and helps dislodge missed stones, and meticulous visual inspection of the operative field with adequate suctioning of any debris, which is particularly critical in patients with acute or gangrenous cholecystitis.

Surgical Management

Once a retained gallstone causes abscess formation or fistulization, definitive surgical intervention is often required. Although minimally invasive or interventional radiology-guided drainage may temporarily control infection, it rarely addresses the root cause, namely, the retained stone [8]. In this patient, the persistent subhepatic abscess and colonic fistula mandated a right hemicolectomy and removal of the offending stone. Such procedures can be done laparoscopically, provided the surgeon is adept in advanced laparoscopic techniques and the patient’s condition is hemodynamically stable. Alternatively, a conversion to an open approach may be necessary when encountering dense adhesions or complex fistulous tracts.

Clinical Implications

This case underscores the importance of considering a retained gallstone in the differential diagnosis of recurrent intra-abdominal sepsis or abscesses following LC. Surgeons and gastroenterologists must maintain a heightened awareness, especially in patients with atypical or unexplained recurrent infections. Early and accurate detection not only prevents multiple hospital admissions but also reduces the morbidity associated with repeated drainage procedures and the potential for more extensive surgical resections.

Moreover, the longer the interval between the initial cholecystectomy and the manifestation of symptoms, the less likely one is to suspect spilled gallstones. Therefore, a thorough operative note detailing any possible intraoperative gallbladder perforation or partial spillage can significantly guide subsequent investigations and avoid protracted diagnostic delays [9].

## Conclusions

Retained gallstones, although infrequent, can pose a significant diagnostic and therapeutic challenge long after a laparoscopic cholecystectomy has been performed. The present case demonstrates how a single spilled stone led to chronic infection, abscess formation, and eventual fistulization into the ascending colon, an outcome that demanded definitive surgical management. This highlights the importance of thorough operative techniques, including meticulous retrieval of any fragmented gallstones and liberal irrigation of the peritoneal cavity. Additionally, clinicians must maintain a high index of suspicion in patients with unexplained recurrent abdominal sepsis or abscesses following gallbladder surgery. Timely recognition and appropriate intervention are critical to minimize morbidity and prevent further complications.

## Appendices

Appendix A 
